# To the Editor

**Published:** 1906-04

**Authors:** F. B. Spooner

**Affiliations:** 6 Halsey St., Brooklyn, N.Y.


To the Editor:

In a New York magazine of December last appears a letter from Dr. Hoffman, of Denver, Colorado. He begins his article thus:

"The subject of state license interchange may be considered in its turn with pyorrhea, and the porcelain as the very latest fad."

Inter change of license means that when a dentist has passed a reputable college, with an additional examination by his home state, he be no more harrassed by other states whose boards may easily be ignorant or corrupt. There are many agitating that one state board should not have the right to try another state, and that such chance for graft, and oppression wiped out, yet Dr. Hoffman calls this a "fad."

In maintaining this attitude the doctor says: "There can be no question of the right and propriety, if the people of any state are willing to accept and feel satisfied with the credentials issued by another state. Neither can there be any question of the right and propriety if the people of any state refuse to accept the credentials of any or of all other states, because of their doubtful value, or on account of local conditions."

The "people" are here referred to twice as responsible for this, when any one that will think one moment will see that the "people" have no knowledge of the niceties of dental law. All such things are originated by interested dentists, and any dental board with an honest desire to interchange, could do so with the greaest *ease.*

All state boards charge fees. In the dark ages pyorrhoea was not known and porcelain was undiscovered. A dissolute king gave to a profligate favorite the right to collect tithes. In time the "people" became alive to this not being *propriety,* and they cut the kings' head off. Colorado takes ten dollars tax. The "people" order it, but the "people" do not get the money. In Italy they have brigands who send a severed ear to the friends of the victim, who must pay a tax, or the man will be killed. The brigands keep faith, hence the saying, "honor amongst thieves." A state board gets the tax in advance, and does not always produce the goods. Nay more, they frequently cut the victim's ears, and tell him to *"try again."*

We now come to *"local conditions."* What interest have the "people" in this? There is nothing "local" in teeth. A man can treat all the teeth in Colorado, if able to do so in New York. He can go on to Japan, also to Egypt, where if he dig up a mummy, he will find that pyorrhoea was a fact not a"fad" as much as six thousand years ago. Yet this doctor that in 1902 was *thought worthy* to examine other dentists (and reject as well) calls pyorrhoea a "fad, the very latest fad."

The doctor says: "In Colorado we have conditions that are radically different from those found in other states. Our geographical location and our climate make the state a convenient stopping place. In addition to its fixed population it is the abode of a transient army which numbers countless dentists many of whom would like for a month or two to practice within our borders to return for every cent in sight, as little work as possible, firm in the belief that our beautifully equipped trans-continental trains would make all their cases entirely successful."

Here we catch a view of "local conditions." Against the chance of bad men coming is the liklihood of good men, those who know something of the treatment of pyorrhoea, and the management of porcelain. What business has an examining board to suspect a man's motives or his honesty. A board sworn to good and truly try the capabilities of a stranger, has no right to think of "trans-continental trains, and every cent in sight."

Dentists do not go to Colorado as transients, they are driven by disease, the desire to prolong their days by breathing mountain air. Surely the good "people" of Colorado would not allow this. Yet Dr. Hoffman says it is the people's will, for he would hardly have the boldness to admit that "local conditions" means private interest against the competition of those who want to escape death."

It may be well to throw light on "local conditions" to relate the writer's experience with the author of this expression. In 1902 the writer interviewed him as to going before the board of Colorado. There I was met with a *stern face*, a *flinty eye*, and obstacles thrown in my way from the very start. I will pass all this to the time when examinations took place. It was in a grand building with a golden dome. Each aspirant was cautioned to write on each paper his name, college, and date of graduation.

Why?

In New York the candidates sign a cipher. Is it proper to surmise in view of the doctors' confession, that it was for the examiners to detect which "were after every cent in sight."

Doctor Hoffman wrote me a letter as follows:

" You failed in four subjects, as well as in practical work, and will have to brush up considerably before coming up again."

Three years after this and I was home in New York, articles began to appear as to the inequity of state boards. I wrote my history in full which appeared in the *"Items"* December, 1904.

Dr. Hoffman's answer in the same medium of February,

1905, was, when boiled down, that the writer was a "liar, and an attempted briber."

In order that the "people" could decide whether I was a scoundrel, or Dr. Hoffman and several of his board deserved this *title,* I prepared a paper. It was an offer of fifty dollars to charity if Dr. Hoffman would produce the *marked papers.* He was to send them to the editor of a New York dental magazine, who with two associates was to decide.

The great dental celebrity registered the challenge, sending it to Dr. Hoffman. He read it as three months later it was returned, but he declined to meet the *shock.*

Here was a secretary who took ten dollars from a New York man, and when offered fifty more would not do several things. Prove their own integrity, and deplete the writer in character and pocket. What a chance, and he did not embrace it.

But a short since the papers were full of labor troubles in Colorado. Scoundrels were murdering others that wanted to work. Gov. Peabody called out the militia, and in a speech said, "all men should have the right to work, and have all the power of the state to protect them." So the militia was called out to deal with the rascals that wanted to dictate, and not a gang of street cleaners was employed to get rid of the state board.

What is the difference?

A labor union is a combination to keep up wages.

A state board (when prostituted) is a combination to keep out other dentists, when "local conditions" indicate. The union invites members, they are honest, they admit they are working for themselves. They blow up enemies with dynamite.

The state board does not invite. It discourages. It does not blow up with explosives, but makes corpses in a quiet way. For it is better to send a man into eternity quick, than to deny him work without which he cannot live. Since the writer met the little man with the "stern face and the flinty eye" there have been mayhap hundreds sifted, and winnowed, and found wanting.

In conclusion I will ask the reader to reflect. When one state board says a man is reputable, and another says disgracefully the reverse, the difference is too great to square with honesty.

"Which were the thieves?"

Finally in whose shoes would the reader be at that grand examination, where envy, sectional feeling, cupidity, and falsehood are as glass. The wretch that fires the bomb, or he who writes a "brush-up" letter, with every cent in sight, and "local conditions" *in his heart.*

F. B. Spooner, D. D. S.

6 Halsey St., Brooklyn, N. Y.

February 20, 1906.

